# Precompensation of 3D field distortions in remote focus two-photon microscopy

**DOI:** 10.1364/BOE.425588

**Published:** 2021-06-01

**Authors:** Antoine M. Valera, Fiona C. Neufeldt, Paul A. Kirkby, John E. Mitchell, R. Angus Silver

**Affiliations:** 1 Department of Neuroscience, Physiology and Pharmacology, University College London, Gower Street, London WC1E 6BT, UK; 2 Department of Electronic and Electrical Engineering, University College London, Malet Place, London WC1E 7JE, UK; 3 These authors contributed equally

## Abstract

Remote focusing is widely used in 3D two-photon microscopy and 3D photostimulation because it enables fast axial scanning without moving the objective lens or specimen. However, due to the design constraints of microscope optics, remote focus units are often located in non-telecentric positions in the optical path, leading to significant depth-dependent 3D field distortions in the imaging volume. To address this limitation, we characterized 3D field distortions arising from non-telecentric remote focusing and present a method for distortion precompensation. We demonstrate its applicability for a 3D two-photon microscope that uses an acousto-optic lens (AOL) for remote focusing and scanning. We show that the distortion precompensation method improves the pointing precision of the AOL microscope to < 0.5 µm throughout the 400 × 400 × 400 µm imaging volume.

## 1. Introduction

Two-photon scanning microscopy is a widely used tool for high resolution functional imaging in life sciences due to its ability to penetrate deep within scattering tissue [1,2]. In neuroscience, two-photon imaging is often used to monitor activity within neurons and across neuronal populations. However, measurements of signals flowing through these 3D structures are restricted with conventional microscopes because the speed of focusing is limited by the inertia of the objective lens. By contrast, rapid remote focus approaches rely on devices that dynamically alter the optical wavefront of the beam prior to the objective, thereby circumventing the need to physically move the objective. Rapid remote focusing has been implemented with electrically tunable lenses (ETLs) [3], liquid crystal spatial light modulators (LC-SLMs) [4–8], phase-locked ultrasound lenses (tunable acoustic gradient (TAG) lenses) [9], deformable mirrors [10], liquid lenses [11], acousto-optic lenses (AOLs) [12–15] and a secondary objective coupled with a piston mirror [16–20]. However, remote focus devices cannot always be positioned in a plane conjugate to the back aperture of the objective, due to mechanical space constraints, a limited range of commercially available focal length lenses and uncertainty in the exact position of the back aperture of commercial objectives. This is problematic because it introduces depth-dependent 3D field distortions in the imaging volume [3,21–24], which impair the performance of remote focus laser scanning systems. Distortions to the 3D field of view (FOV) lead to missed regions of the specimen and serious mismatches between the 3D *z*-stacks obtained by mechanically moving the objective and the *z*-stacks obtained by remote focusing. It also causes erroneous measurements of the dimensions of biological features [21] and makes selection and positioning of regions of interest (ROIs) for selective imaging or photostimulation, within the full FOV inaccurate, which is particularly detrimental when targeting small structures such as neuronal processes.

In a conventional microscope with a collimated input and an infinity corrected objective, non-telecentricity has little effect on the 2D FOV when the focus is changed by mechanically shifting the objective. Although a lateral misalignment of the input beam will result in a tilted point spread function (PSF), changing the focus will not introduce *xy* plane magnification distortions. However, in a remote focus microscope, where the curvature and tilt of the input beam is modified to change the 3D focal position, a misalignment of the optical input from its ideal telecentric position leads to a distorted 3D FOV. An axially misaligned optical input leads to a *z*-dependent *xy* magnification, as well as a varying axial magnification, resulting in uneven *z*-plane separation. A lateral misalignment of the remote focus unit results in a skew distortion to the *xz* and/or *yz* FOV. As the laser is focused in *z* away from the natural plane (the focal plane for a planar input beam), the FOV progressively drifts laterally. This is problematic as even modest misalignments of the remote focus unit can result in distortions of the dimensions of the FOV and lateral shifts amounting to tens of micrometers over the focus range.

Previous distortion correction approaches [21] have been implemented via post-acquisition image processing. This has several limitations, including loss of information due to cropping of the volumetric data, the time-consuming nature of *post-hoc* corrections and the use of a complex calibration sample that requires laser manufacturing and which is sensitive to misalignments. Here, we present a fast, convenient, and generic method to measure and precompensate distortions in remote focus microscopes. We demonstrate experimentally that a paraxial remote focus distortion model can be applied to an AOL remote focus microscope to precompensate for the 3D field distortions arising from non-telecentric misalignments and show how similar distortion precompensation solutions could be applied to other remote focus technologies.

## 2. Model of 3D field distortions in remote focus microscopes

**Fig. 1.**
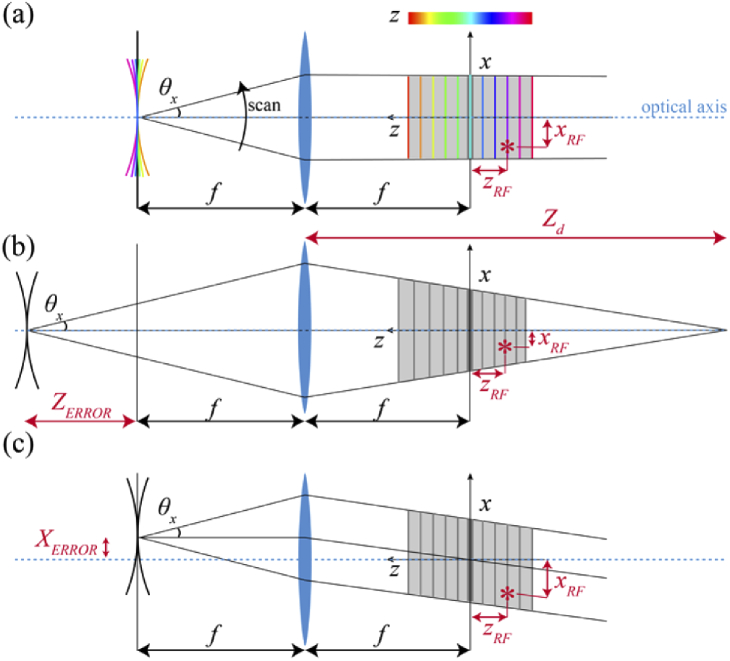
**Telecentricity in remote focus microscopes**. **a)** Schematic ray diagram showing *xz* plane for telecentric output of the wavefront shaping unit located one focal length (*f*) from the imaging objective (*blue* lens). Curved wavefronts (*curved rainbow-colored* lines) represent a converging or diverging optical beam, being scanned (curved arrow). Central ray (*black* lines) deflected through a beam semi-scan angle *θ_x_*, with respect to the optical axis (*dotted blue* line). Each of the three central rays indicate a different time point of a scan. *Red asterisk* shows the beam focused to an arbitrary *xz* remote focus within the FOV (*gray* area) with coordinates (*x_RF_, z_RF_*) indicated. The change in the axial focus of the objective is linearly proportional to the change in curvature produced by the remote focus unit and the central ray in the FOV has no skew distortion. Imaging planes (*straight rainbow-colored* lines) are thus equally spaced in *z* for equal steps in beam curvature and the FOV is of constant size. Note that the rainbow color-code matches [Fig. 3–5]. **b)** Axial misalignment of the remote focus device output (*Z_ERROR_*) causes depth-dependent *xy* FOV magnification and a non-constant *z*-plane spacing with wavefront curvature, resulting in a change in both *x_RF_* and *z_RF_* with respect to the telecentric case in (a). *Z_d_* is the position of the plane conjugate with the output of the remote focus device. **c)** Lateral misalignment of the remote focus device output (*X_ERROR_*) introduces a skew distortion to the FOV, resulting in a skewed local *z*-axis and a shifted *x_RF_*.

To characterize and correct for the 3D field distortions that arise in a remote focus microscope due to non-telecentric misalignments of the remote focus unit, we developed a paraxial model of a remote focus unit and objective lens. In an ideal telecentric case [Fig. 1(a)] the position of the output of the remote focus unit is located one focal length distance away from the objective (i.e. conjugate to the back aperture) and the optical curvature change produced by the remote focus unit is linearly proportional to the resulting change of *z-*remote-focus (*z_RF_*) in the FOV. Note that *z_RF_* is measured with respect to the natural focal plane and is considered a negative distance when located below the natural focal plane (as is the case in Fig. 1). Since the plane conjugate to the output of the remote focus unit is located at infinity, the magnification of the *xy* FOV is constant with changes in axial focus. An axial misalignment of the remote focus unit, *Z_ERROR_*, results in a *z*-dependent *xy* magnification of the objective FOV, together with a non-uniform *z*-plane separation for equal changes in curvature of the optical input [Fig. 1(b)]. In this case, the change in position of *z_RF_* is not proportional to the change in optical curvature produced by the remote focus device and the resulting *z*-dependent *xy* magnification causes tapering of the *x* FOV with *z*. A lateral displacement in *x, X_ERROR_*, of the output of the remote focus unit results in a laterally shifted position of the *x*-remote-focus (*x_RF_*) with *z*, which we refer to as an *xz* skew distortion of the imaging volume [Fig. 1(c)]. Similarly, a lateral offset in *y* would cause a *yz* skew distortion.

To quantify the field distortions arising from non-telecentric misalignments of the remote focus unit we derived a set of equations (see Supplement 1, Section S1 and [Fig. S1] for the full derivation). For an optical output of curvature *κ*, a paraxial objective lens of focal length *f*, an imaging refractive index *n* and an (*x*, *z*) misalignment of the remote focus unit (*X_ERROR_*, *Z_ERROR_*), the (*x*, *z*) remote focus with respect to the optical axis, (*x_RF_*, *z_RF_*) is given by,

(1) $$z_{RF}\;=\;\frac{-n\;f^{2}\;\kappa }{\kappa \;Z_{ERROR}\;+\;\text{1}}$$

(2) $$x_{RF}\;=\;\frac{f(\theta_{x}\;-\;\kappa \;X_{ERROR})\;}{\kappa \;Z_{ERROR}\;+\;\text{1}}$$

where *θ_x_* is the uncompensated *x* semi-scan angle. Note that Fig. 1 shows the deflection of the central ray of the wavefront at the beginning, middle and end of a scan. For an axially misaligned remote focus unit, the lateral *xy* plane magnification *M*, will vary with *z* and is given by,

(3) $$M\;=\;\frac{nf^{2}\;+\;z_{RF\;}Z_{ERROR}}{nf^{2}}$$

To precompensate for the distortion, the remote focus unit can be driven to produce a wavefront curvature *κ_COMP_* and *x* semi-scan angle *θ_x COMP_*, based on the inverse of Eqs. (1) and (2), for the desired corrected *z* and *x* focus in the objective FOV, *z_CORR_* and *x_CORR_*, respectively, where

(4) $$\kappa_{COMP}\;=\;-\;\frac{z_{CORR}}{\;z_{CORR\;}Z_{ERROR}\;+\;nf^{2}\;}$$

(5) $$\theta_{x\;COMP}\;=\;\kappa_{COMP\;}X_{ERROR}\;+\frac{x_{CORR}\;(\kappa_{COMP\;}Z_{ERROR}\;+\text{1})}{f}\;$$

Thus, field distortions arising from system non-telecentricity can be perfectly compensated, if the wavefront curvature *κ* and (*x, y*) semi-scan angles (*θ_x_*, *θ_y_*) produced by the remote focus unit are known. The generic nature of this solution suggests that field distortions caused by any paraxially approximated remote focus system can be precompensated by incorporating Eqs. (4) and (5) into the control software.

**Fig. 2.**
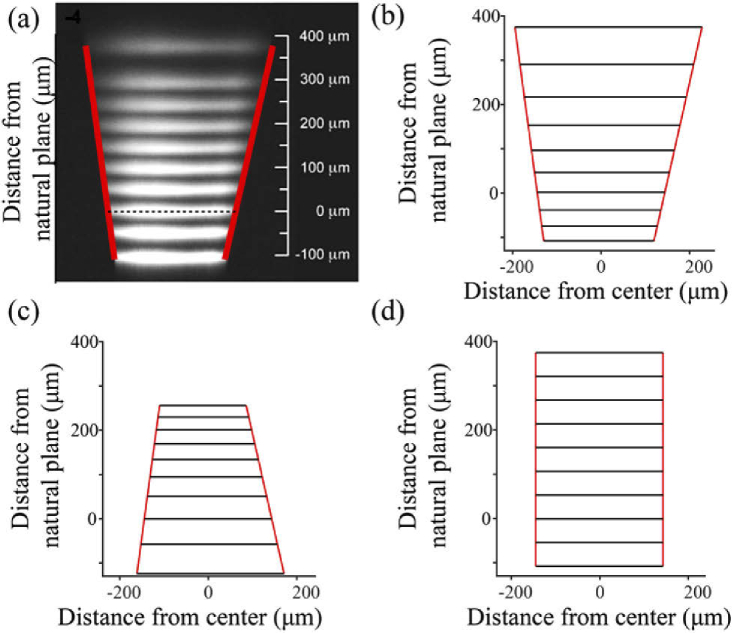
**Application of the distortion model to an electrically tunable lens remote focus system. a)** Experimental *xz* FOV imaged in fluorescein beneath the objective of a two-photon microscope, remote focused with an electrically tunable lens. Adapted with permission from [3] © The Optical Society. *Red* lines show extent of *x* limits of the FOV predicted using eqs. (1,2) with *f* = 4 mm, *Z_ERROR_ *= 26.6 mm, *X_ERROR_* = 0.4 mm, *n* = 1.33 and *θ_x_*_ _= ± 36 mrad. Natural focal plane (*z* = 0) indicated by *dashed black* line. **b)** Theoretical *xz* FOV for a *z* range = [−108, 375] µm with 9 equal steps of curvature, given eqs. (1,2). **c)** Theoretical *xz* FOV calculated using precompensated drives *κ_COMP_*, Eq. (4) and *θ_x COMP_*, Eq. (5). **d)** Accurately precompensated rectangular FOV with equispaced *z* planes calculated by driving the non-telecentric model of the remote focus system of (a) and (b) with the pre-compensated drive of (c).

Magnification distortions are common in widely used ETL-based remote focus microscopes because they are often placed in a non-telecentric position behind the objective [22–24]. As a worked example, we show how our solution could be used to precompensate the remote focus distortion reported in an ETL-based non-telecentric remote focus two-photon microscope [3]. Figure 2(a) shows the reported FOV and 2(b) the FOV fitted by Eqs. (1,2). The precompensated FOV calculated by Eqs. (4, 5) is shown in Fig. 2(c). By driving the non-telecentric remote focus unit using Eqs. (4, 5), a perfectly undistorted FOV would be obtained, as predicted in Fig. 2(d).

## 3. Experimental evaluation of the distortion precompensation scheme

**Fig. 3.**
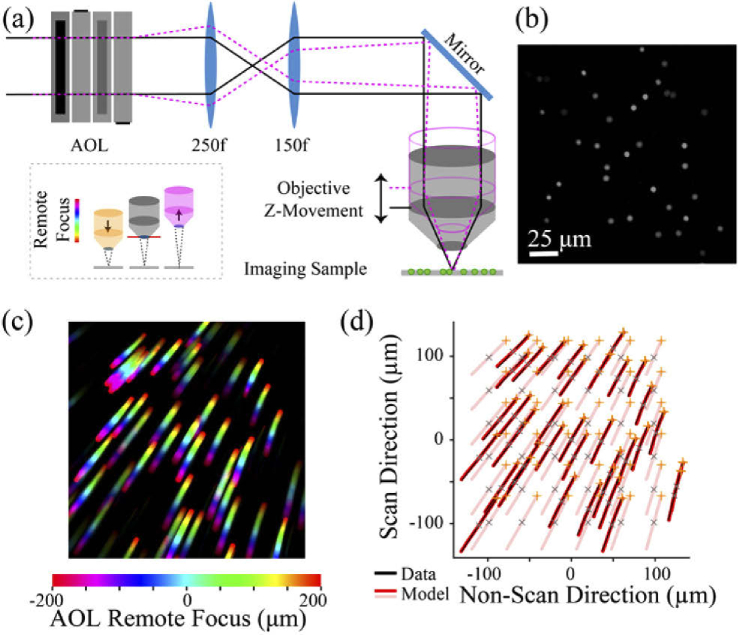
**Characterization of non-telecentric remote focus distortions in an acousto-optic lens microscope**. **a)** Experimental setup, showing a slide covered with a thin layer of 5 µm green beads. As the axial position of the objective is incrementally shifted away from the sample using motorized *z*-movement, the beads are kept in focus using remote focusing with an acousto-optic lens (AOL) microscope. *Black solid* line shows optical beam focusing at the natural plane (*gray* objective). *Pink dotted* line shows converging optical beam enabling remote focused imaging with objective displaced (*pink* outlined objective). *Inset* summarizes the experimental procedure used to generate panel (c). **b)** Reference image of 5 µm fluorescent beads obtained at the natural focus of the objective in a 262 µm *xy* FOV. **c)** Color-coded intensity projection of the imaged beads at various remote foci (± 200 µm in 5 µm increments, color indicates *z*-plane location). Color-coded lines indicate depth-dependent deviation of the *xy* bead location relative to the natural plane image. **d)** Bead trajectories from (c) in scanned (*y*) and non-scanned (*x*) direction tracked using ImageJ TrackMate plugin (*black*). Bead trajectories predicted from the model at the same location as the experiments (*red*) and for positions on a grid pattern (*pink*). *Orange* and *gray* asterisks indicate location of beads for +200 µm remote focusing and natural plane imaging, respectively.

To test the accuracy of distortion precompensation we developed a simple strategy [Fig. 3(a)] to measure field distortions and employed an acousto-optic lens (AOL) remote focus microscope [Fig. S2] (Supplement 1 Section S2), since AOL technology enables high precision focusing and scanning of a laser beam [25,26]. To quantify distortions caused by non-telecentric remote focusing in our AOL 3D two-photon microscope, we compared a reference image obtained at the natural plane of the objective [Fig. 3(b)], where non-telecentric misalignment does not cause distortion of the FOV, with images of the same region obtained using increasingly strong remote focusing. The resulting change in focus was systematically counteracted by displacing the objective by an equal amount in the opposite direction [Fig. 3(a), inset]. The precision of the mechanical objective focus, which is critical to the precision of the calibration scheme, was determined to be <0.5 µm, with a mechanical alignment gauge.

To perform the calibration, we used a microscope slide with a thin layer of 5 µm fluorescent beads suspended in agar (Phosphorex, Degradex), although any small, bright sample would work (see Supplement 1 Section S3 and [Fig. S3]). In a perfectly telecentric optical path, the mechanical objective focus and AOL remote focus would be in perfect registration and the images of the beads would remain superimposed wherever they were acquired across the entire remote focusing *z*-range. However, when non-telecentric misalignment is present, field distortions are revealed as displacements in the locations of the beads in the images across the *z*-range. Figure 3(c) shows a *z*-intensity projection of the images of the calibration *z*-stack (*C*-*z*-stack) covering a remote focus range of ± 200 µm, without correction for the distortion arising from the misalignments present in our AOL 3D two-photon microscope. Displacement of the individual beads across the *C*-*z*-stack were measured using the TrackMate plugin in ImageJ [27]. When plotting the position of the beads for each frame of the *C*-*z*-stack, the trajectory of the displacement of each bead over the remote focus *z*-range enables a measure of the distortion field [Fig. 3(d)]. The *X_ERROR_* and *Y_ERROR_* present in our AOL microscope led to a measured absolute shift in *x_RF_* and *y_RF_* of 28 µm and 14 µm, respectively, with respect to the undistorted *x_RF_* and *y_RF_* at *z* = 0, over a remote focus range of ± 200 µm. The *Z_ERROR_* present in our AOL microscope caused the reference 267 × 267 µm *xy* FOV to vary between 252 × 252 µm and 283 × 283 µm over a ± 200 µm remote focus range, respectively.

To relate bead trajectories to non-telecentric field distortion in a quantitative manner we developed a ray-based model of the AOL 3D microscope. The non-telecentric remote focus distortion predicted by the AOL ray model was found to match the distortion predicted by the paraxial distortion model [Fig. S4-S6]. The ray model was then used to estimate the values of non-telecentric misalignment in the physical system that give rise to the observed distortion, by finding a match between the experimentally measured distortion and the distortion predicted by the model. For the experimental data in Fig. 3(d), the distortion predicted by the model is likely to be caused by a misalignment of the AOL scanner of *X_ERROR_*, *Y_ERROR_*, *Z_ERROR_* of 2.3, 1.3, -69 mm, respectively [Fig. S7]. But note that the same experimental field distortion could be caused by a wide range of optical component misalignments in the relay(s) between the remote focus device and the final objective. Nevertheless, the predictions of Eqs. (1) and (2) will still be valid. We then used Eqs. (4,5) to precompensate for the distortion, by modifying the amount of tilt and curvature imparted by the AOL to the optical beam. The distortion precompensation equations were incorporated into our MATLAB-based AOL microscope control software and had little computational overhead. The values for *X_ERROR_, Y_ERROR_* and *Z_ERROR_* were adjusted to obtain a perfect registration between the remote focus system and the mechanical focus system.

**Fig. 4.**
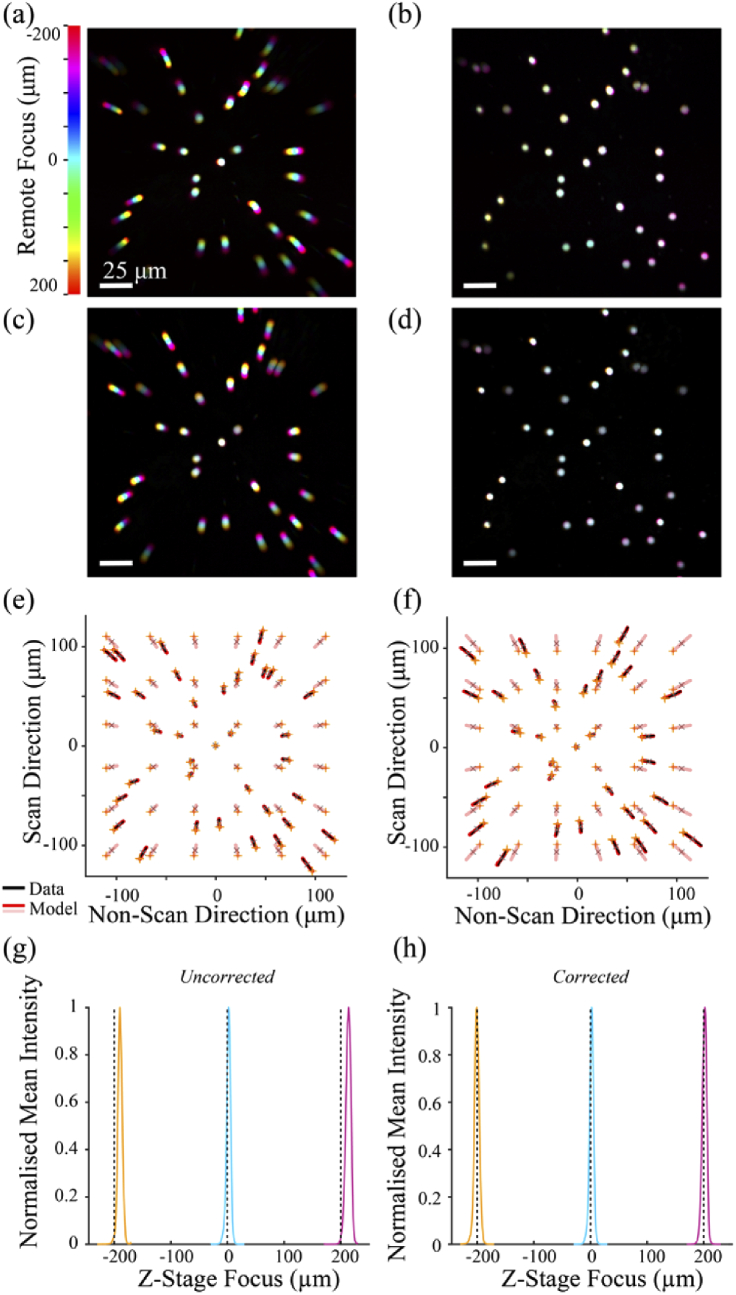
**Experimental tests of distortion correction scheme for axial AOL misalignments: a)** Mean intensity projection of imaged beads at various remote foci (± 200 µm in 5 µm increments, color indicates *z*-plane location). Color smearing indicates depth-dependent deviation of the bead location relative to the natural plane image. The −83 mm of additional effective axial misalignment (see Fig. S8(b)) caused *z*-dependent magnification of the FOV (lateral misalignment was already pre-compensated). **b)** Same as (a) with axial distortion precompensation enabled. **c)** Same as (a), with introduction of +60 mm of additional axial AOL misalignment (See Fig S8(c)). **d)** same as (c) with distortion precompensation enabled. **e)** Bead position trajectories tracked using ImageJ TrackMate plugin (*black*) and bead positions predicted by the ray model at the same locations (*red*) together with model predictions for a grid pattern (*pink*) for (b), with *Z_ERROR_* = +60 mm. *Orange* and *gray* asterisks indicate values for +200 µm remote focusing and natural plane imaging, respectively. **f)** same as (e), with *Z_ERROR_* = −83 mm. **g)** Measure of the *z*-focus error in our AOL system when remote focusing at +200 and +200 µm, using 0.2 µm beads. Out-of-focus error was −9.5 and +13.5 µm, respectively. **h)** Correction for axial misalignment also corrected non-constant spacing of the *z* planes. Residual out-of-focus error is −1.5 and +1.5 µm.

To test the performance of precompensation for magnification distortions arising from axial misalignments of the AOL scanner, the amount of effective axial misalignment, *Z_ERROR_*, was varied by changing the lenses within the relay, which altered the magnification and introduced an effective axial misalignment of 152 mm or −9 mm (i.e. an addition of −83 mm or +60 mm to the pre-existing axial misalignment of −69 mm in our AOL setup, respectively) [Fig. S8]. Figure 4(a-f) shows the projection before and after the correction of these effective axial misalignments, together with model predictions, confirming the capacity of the distortion precompensation scheme for larger axial misalignments of the remote focus unit. In addition, the *z* focal plane position was measured before and after distortion precompensation [Fig. 4(g,h)], using a thin layer of 0.2 µm beads suspended in polymer. The AOL was used to remote focus at *z* = [−200, 0, 200] µm, and the objective was mechanically moved in steps of 1 µm around these positions to measure the real amount of remote focusing. Before precompensation, the out-of-focus error was measured to be −9.5 µm and 13.5 µm for a target remote focus of +200 µm and 200 µm, respectively, but was reduced to only −1.5 µm and 1.5 µm, after precompensation. This confirmed that our precompensation for axial misalignment also corrected for the non-constant *z*-spacing introduced by the pre-existing axial misalignment of −69 mm.

**Fig. 5.**
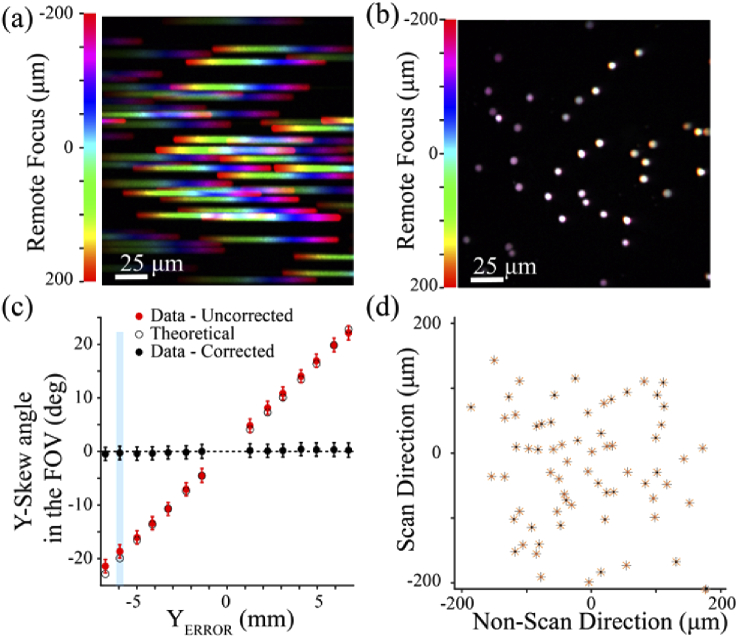
**Lateral distortion precompensation: a)** Color-coded maximal intensity projection of fluorescent beads for a ±200 µm remote focusing range with 5 µm *z*-increments, without correction and with the beam incident on the back aperture of the objective misaligned using the Risley prisms (equivalent to a −5.9 mm *Y_ERROR_*). **b)** Same as (a) with distortion precompensation. **c)** YZ skew distortion angle in the FOV of the objective (arctan of the *yz* slope of the central bead) as a function of the effective lateral AOL misalignment in *y* (*red* symbols). The distortion due to the inherent axial misalignment of the AOL was precompensated using the correction scheme to isolate the distortion arising from the Risley prism beam deflection (*black* symbols). Theoretical skew distortion angle measurements (*hollow black* circles). *Light blue* area indicates the experimental point shown in (b, d). **d)** Trajectories of the bead positions (*black*) with both axial and lateral precompensation. *Orange* and *gray* asterisks indicate location of beads values for +200 µm remote focusing and natural plane imaging, respectively. Note that markers and lines are superimposed as residual error is only 0.3 ± 0.2 µm.

The performance of the distortion precompensation scheme was tested for a range of effective lateral misalignments of the AOL by deflecting the beam with a pair of Risley prisms (PS810-B, Thorlabs Inc.). This allowed us to introduce a lateral AOL misalignment in a controlled manner, at a high precision, without having to physically move the AOL [Fig. S9]. The effective lateral misalignment of the AOL, *X_ERROR_* and *Y_ERROR_* could be calculated from the lateral displacement of the optical beam by the Risley prisms and the magnification of the optical telecentric relay. To assess the performance of the distortion correction scheme, the *xz* and *yz* skew distortion of the FOV was measured by taking the inverse tangent of the slope of the trajectory of the beads through the *C*-*z*-stacks, with and without the distortion correction scheme enabled, for a range of lateral beam displacements. Figure 5(a,b) shows the maximum intensity *z*-projections of the *C*-*z*-stacks acquired without and with distortion precompensation, for an effective lateral AOL misalignment of −5.9 mm in *y*. Despite the strong distortion introduced by the *y* misalignment (−18.7 ± 1.3 degree *yz* skew angle over a ± 200 µm remote focus range), the distortion precompensation reduced the *yz* skew angle to −0.3 ± 1.3 degrees. Risley prism-based lateral beam displacement across a range of values showed that the distortion compensation scheme was effective at correcting skew distortions arising from effective lateral displacements of the remote focus unit of up to ± 6.8 mm in *y* [Fig. 5(c)]. Similar results were obtained for *x* displacement.

To quantify the precision of AOL-based laser scanning throughout the 400 × 400 × 400 µm imaging volume after distortion precompensation, we measured the largest displacement of the bead from its position when imaged at the natural focal plane of the objective (*z* = 0). Figure 5(d) provides an example of the bead displacement measured over a *C*-*z*-stack, *after* correction for the inherent lateral and axial misalignments of the AOL. This revealed a maximum lateral displacement of 0.33 µm ± 0.12 µm over the ± 200 µm remote focus range.

An unexpected benefit of the high precision of the distortion precompensation scheme for non-telecentric misalignments enabled us to identify and correct for small residual distortions in our AOL microscope that arose from weak divergence of the input beam [Fig. S10]. This higher order distortion, which resulted in a non-uniform distortion of the field with a scan-time dependency, could be explained by the ray model (Supplement 1 Section S6), enabling a full correction. This was possible only after precompensation for the larger first order distortions due to system non-telecentricity.

## 4. Discussion and conclusion

Wavefront-shaping devices are increasingly being used in two-photon microscopy to provide the rapid focusing required for 3D functional imaging and 3D photostimulation. Here we derive a set of equations to quantify and correct for the substantial field distortions introduced by remote focus devices that are not perfectly telecentric. By quantifying distortions and implementing distortion precompensation into the control software of an AOL-based remote focus 3D two-photon microscope, we demonstrated submicron (< 0.5 µm) precision over the 400 × 400 × 400 µm FOV, for a non-telecentric arrangement. The generic nature of the precompensation solution makes it suitable for a range of remote focus devices, enabling non-telecentric designs with improved optical performance.

Addition of remote focusing devices to conventional microscopes is complicated by the requirement for telecentricity within the optical train [28], which is often not possible due to mechanical space constraints, lens availability and a lack of information on the optical design of commercial microscopes and objectives. The substantial field distortions introduced by non-telecentric arrangements [3,21–24] compromise the imaging performance of such 3D microscopes. Our solution to this problem, which enables full precompensation of distortion over the entire 3D FOV, builds on earlier work that quantified remote focus distortions using a sophisticated 3D plastic reference calibration specimen and an image processing based on *post-hoc* correction [21]. Our quantification method and precompensation strategy has several advantages over such an approach. The simple *C-z*-stack calibration method using fluorescent beads dispenses with the need for a custom made 3D calibration sample, and thus any issues associated with the polymer having a different refractive index to tissue and hence a different distortion (as noted by the authors, [21]). By contrast, our calibration and precompensation can be carried out in water or even in the target biological tissue itself. This allows any optical element located between the lens and the sample, such as a coverslip, to be integrated into the calibration process. Moreover, our approach directly calibrates the microscope against the *xyz* stage mechanics and thus provides a single coordinate system for the 3D FOV of the specimen when zooming and/or imaging in any arbitrary plane. Lastly, our precompensation solution restores the full telecentric imaging volume, so that remote focusing and mechanical focusing are in perfect register and laser scanning in any arbitrary direction maps accurately onto a Euclidean 3D space. This prevents regions being missed and eliminates the necessity of post processing the 3D images.

Quantification and precompensation of field distortions, represents a significant step towards achieving aberration-free non-telecentric remote focusing. Compensation for field distortions does not, however, correct for the (largely spherical) aberrations introduced by the objective lens when remote focusing. These have recently been characterized in detail and compensated for in a LC-SLM-remote focus microscope [29]. Combining field and lens-based aberration corrections would enable non-telecentric remote focus designs to approach the near optimal optical performance achieved with telecentric dual objective 3D microscopes [16,20].

Precompensation of remote focus field distortion improves the performance of AOL-based random-access 3D microscopy in several ways. Firstly, it ensures that the location and extent of the FOV is the same as with a perfectly telecentric optical arrangement. The sub 0.5 µm precision achievable with AOL-based laser scanning is critical for selective high-speed functional imaging of fine biological structures such as the synapses, axons and dendrites of neurons. High precision line scanning within an undistorted imaging volume is also critical for the correction of tissue movement in real time, since this involves tracking the movement of an object such as a fluorescent bead, in one location and applying a rigid *xyz* translation to the imaged volume, to counteract any 3D brain movement [30]. Lastly, an undistorted remote focus FOV enables highly precise alignment of neighboring AOL-based *z*-stacks for imaging large biological structures and brain regions that extend far beyond a single imaging volume.

As our modelling of ETL remote focusing shows, our approach for correcting distortions in non-telecentric remote focus devices is applicable to other types of 3D microscopes and is expected to improve their performance. For remote focus technologies in which the wavefront shaping control is less precise than that of an AOL or ETL, some *post-hoc* image processing may still be required. However, if the non-telecentric remote focus distortions are largely precompensated, the loss in volumetric data will be significantly reduced and any residual errors may be corrected with post-processing of the images. Alternatively, the estimate of the non-telecentric misalignments provided by the distortion model can be used to correct the misalignment of optical components in the remote focus path. The advantage of this is that it will also correct misalignment-induced aberration of the PSF.

Precompensation for field distortions is also likely to be particularly important for 3D photostimulation, since this requires accurate focusing to one or more precise locations within the imaging volume. Indeed, 3D two-photon imaging and optogenetic photostimulation methods [7,31–34] require accurate LC-SLM-remote focusing of multiple illumination beams to specific locations within the imaging volume, which is typically acquired with galvanometer-based 2D scanning and mechanical focusing. Precise precompensation of the imaging field will be critical for extending these technologies to combine full 3D random access functional imaging and 3D photostimulation and for making photostimulation more precise, so that smaller structures such as synapses (∼1µm) can be selectively photoactivated.

## Data Availability

The SilverLab AOL Imaging Software and scripts for models are available on GitHub [35]. Data underlying the results presented in this paper are available on FigShare [36].

## Supplemental document

See Supplement 1 for supporting content.
