# Supplementary material for: Precompensation of 3D field distortions in remote focus two-photon microscopy
Source: Biomed Opt Express. 2021 Jun 1;12(6):3717–28. doi: 10.1364/BOE.425588 (PMC8221938; doi:10.1364/BOE.425588)
Supplement: Supplementary file 1 [file boe-12-6-3717-s001.pdf]

Supplemental Document

Biomedical Optics EXPRESS

Precompensation of 3D field distortions in remote focus two-photon microscopy: supplement

Antoine M. Valera,[1,3] Fiona C. Neufeldt,[1,2,3] Paul A. Kirkby,[1] John E. Mitchell,[2] and R. Angus Silver[1,*]

[1]*Department of Neuroscience, Physiology and Pharmacology, University College London, Gower Street, London WC1E 6BT, UK*
[2]*Department of Electronic and Electrical Engineering, University College London, Malet Place, London WC1E 7JE, UK*
[3]*These authors contributed equally*
[*]*a.silver@ucl.ac.uk*

This supplement published with The Optical Society on 1 June 2021 by The Authors under the terms of the Creative Commons Attribution 4.0 License in the format provided by the authors and unedited. Further distribution of this work must maintain attribution to the author(s) and the published article's title, journal citation, and DOI.

Supplement DOI: https://doi.org/10.6084/m9.figshare.14597292

Parent Article DOI: https://doi.org/10.1364/BOE.425588

Precompensation of 3D field distortions in remote focus two-photon microscopy: supplementary notes

ANTOINE M. VALERA,[1,†] FIONA C. NEUFELDT,[1,2,†], PAUL A. KIRKBY[1], JOHN E. MITCHELL[1] AND R. ANGUS SILVER[1,*]

[1] Department of Neuroscience, Physiology and Pharmacology, University College London, Gower Street, London WC1E 6BT, UK
[2] Department of Electronic and Electrical Engineering, University College London, Malet Place, London WC1E 7JE, UK
**a.silver@ucl.ac.uk*
† These authors contributed equally.

Abstract: This document contains supplementary information to "Precompensation of 3D field distortions in remote focus two-photon microscopy". It provides the derivation of the equations describing the non-telecentric distortion model (section S1) and includes details on the optical path of an acousto-optic lens (AOL) remote focus two-photon microscope (section S2). An outline of the method of implementing distortion precompensation with a single fluorescent bead is given in section S3. Section S4 gives examples of the 3D field distortions predicted by the ray model for a non-telecentric AOL microscope and shows the experimentally observed distortion before correction. Experimental methods are described in section S5 and details on the correction of higher order field distortions after compensation for the non-telecentric distortions are given in S6.

© 2020 Optical Society of America under the terms of the OSA Open Access Publishing Agreement

S1. Precompensation of the remote focus distortions

This section outlines the equations describing the distortion model and the correction method. In a remote focus system, the z-focus is controlled by the wavefront curvature κ imparted to the beam by the remote focus unit. For $\kappa = 0$, the beam incident on the objective will be planar, resulting in a focus in the natural focal plane of the objective, at a distance one focal length from the objective. For a positive or negative κ, the objective input beam diverges [Fig. S1(a)] or converges [Fig. S1(b)]. This is shown in Fig. S1 (a) and (b) for a single time point, where the diverging or converging wavefront results in a remote z-focus below or above the natural focal plane, respectively. The z-remote-focus in the field of view (FOV), z_{RF} is the distance of the focus from the natural focal plane of the objective (i.e. when $\kappa = 0$) and may be found by applying the thin lens equation,

$$\frac{1}{f} = \frac{1}{u} + \frac{n}{v} \tag{S1}$$

where f is the focal length of the paraxial objective, u is the distance from the lens to the convergence point of the output of the remote focus unit, v is the distance from the lens to the remote focus produced in the FOV and n is the refractive index of the imaging medium. Applying equation S1 to the non-telecentric system shown in Fig. S1, where Z_{ERROR} is the axial misalignment of the remote focus unit from its telecentric position, gives $u = (f + Z_{ERROR} + 1/\kappa)$ and $v = (nf - z_{RF})$ and solving for z_{RF} gives:

$$z_{RF} = \frac{-nf^2\kappa}{\kappa\, Z_{ERROR} + 1} \tag{S2}$$

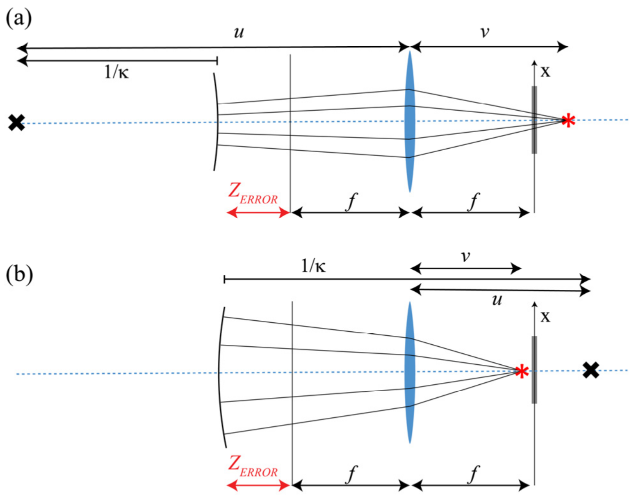

Fig. S1. Remote focus for an axially misaligned remote focus unit. Remote focus unit axially misaligned by an amount Z_{ERROR} and objective lens of focal length f. **(a)** The diverging optical output of the remote focus unit results in a remote focus (*red* asterisk) below the natural focal plane of the objective (*gray* plane). The curved wavefront (*black* arc) converges at a point (*black* cross) a distance $1/\kappa$, where κ is the curvature of the wavefront in m^{-1}. u is the distance from the point of convergence to the objective lens and v is the distance from the objective lens to the remote focus z_{RF}. **(b)** Same as (a) but for a converging optical output, resulting in a remote focus above the natural focal plane.

From this it can be seen that in the case of an axially misaligned remote focus unit, z_{RF} is no longer proportional to the wavefront curvature κ.

In Fig. 1 of the main text, Z_d is the distance between the objective lens and the plane conjugate to the output of the remote focus unit, which can be found by applying equation S1:

$$Z_d = \frac{nf^2 + fZ_{ERROR}}{Z_{ERROR}} \tag{S3}$$

Moreover, the magnification, M in the xy plane is given by

$$M = \frac{Z_d - nf + z_{RF}}{Z_d - nf} \tag{S4}$$

and substituting the expression for Z_d given by eq. S3, M can then be expressed as

$$M = \frac{nf^2 + z_{RF}Z_{ERROR}}{nf^2} \tag{S5}$$

For the case of a remote focus unit misaligned in (x,z) by an amount (X_{ERROR}, Z_{ERROR}), the position of the focus in the x-plane, x_{RF}, as measured from the optical axis depends on the projection of X_{ERROR} along the tilted local z-axis and the varying lateral magnification due to Z_{ERROR} and is given by:

$$x_{RF} = X_{ERROR}\frac{z_{RF}}{nf} + \theta_x(f+Z_{ERROR})\left(\frac{Z_d - nf}{Z_d}\right)M \tag{S6}$$

where θ_x is the x semi-scan angle of the beam by the remote focus unit. Substituting the expressions for Z_d and M given by equations S3 and S4, x_{RF} may be rewritten as

$$x_{RF} = \frac{f(\theta_x - \kappa X_{ERROR})}{\kappa Z_{ERROR} + 1} \tag{S7}$$

Similarly, the y-focus in the FOV is given by,

$$y_{RF} = \frac{f(\theta_y - \kappa Y_{ERROR})}{\kappa Z_{ERROR} + 1} \tag{S8}$$

where θ_y is the y semi-scan angle.

Equations S2, S7 and S8 describe the distorted 3D focal position (x_{RF}, y_{RF}, z_{RF}) produced in the FOV, for a remote focus unit misaligned in (x,y,z) by an amount (X_{ERROR}, Y_{ERROR}, Z_{ERROR}). By rearranging these equations for κ, θ_x and θ_y, an expression can be found for the wavefront curvature and beam semi-scan angle that precompensates for the distortion, for a corrected (x,y,z) focus at (x_{CORR}, y_{CORR}, z_{CORR}). These expressions, κ_{COMP}, $\theta_{x\ COMP}$ and $\theta_{y\ COMP}$, are integrated into the control software of the AOL and are given by,

$$\kappa_{COMP} = \frac{-z_{CORR}}{z_{CORR} Z_{ERROR} + nf^2} \tag{S9}$$

$$\theta_{x\,COMP} = \kappa_{COMP} X_{ERROR} + \frac{x_{CORR}(\kappa_{COMP} Z_{ERROR} + 1)}{f} \tag{S10}$$

$$\theta_{y\,COMP} = \kappa_{COMP} Y_{ERROR} + \frac{y_{CORR}(\kappa_{COMP} Z_{ERROR} + 1)}{f} \tag{S11}$$

For remote focus systems containing a telecentric relay of magnification M_R between the remote focus unit and objective, the expressions for κ_{COMP}, $\theta_{x\ COMP}$ and $\theta_{y\ COMP}$ are given by,

$$\kappa_{COMP} = \frac{-z_{CORR} M_R^2}{z_{CORR} M_R^2 Z_{ERROR} + nf^2} \tag{S12}$$

$$\theta_{x\,COMP} = \kappa_{COMP} X_{ERROR} + \frac{x_{CORR} M_R (\kappa_{COMP} Z_{ERROR} + 1)}{f} \tag{S13}$$

$$\theta_{y\,COMP} = \kappa_{COMP} Y_{ERROR} + \frac{y_{CORR} M_R (\kappa_{COMP} Z_{ERROR} + 1)}{f} \tag{S14}$$

S2. Optical path of the remote focus acousto-optic lens 3D microscope

Fig. S2 shows the optical setup of the acousto-optic lens (AOL) 3D two-photon microscope. A femtosecond pulsed laser (2 W at 920 nm; Chameleon Ultra II, Coherent Inc.) passes through a double pass prism-based pre-chirper before reaching the AOL 3D scanner. The custom-designed prism-based pre-chirper (APE GmbH, Berlin) introduces a group velocity dispersion of approx. 29,000 fs^2 to compensate for the temporal dispersion introduced by the AOL and the optical lenses in the system. The AOL consists of two orthogonally arranged pairs of TeO_2 AODs (Gooch and Housego) and comprises a compact geometry [1,2]. The AODs are interleaved with quarter-wave plates and polarizers to couple the beam into the subsequent AODs and to block the unwanted zero-order beam. The omission of inter-AOD telecentric relays that are included in earlier AOL designs [3,4], reduces the path length of the AOL from approx. 1.5 m to 20 cm. The AOL scanner deflects and adds curvature to the optical beam, which is subsequently relayed to the back focal plane of the objective lens by a relay lens system. The upright microscope consisted of an in-house optical arrangement mounted on top of a SliceScope (Scientifica, UK) with a telecentric relay, arranged to underfill a water-

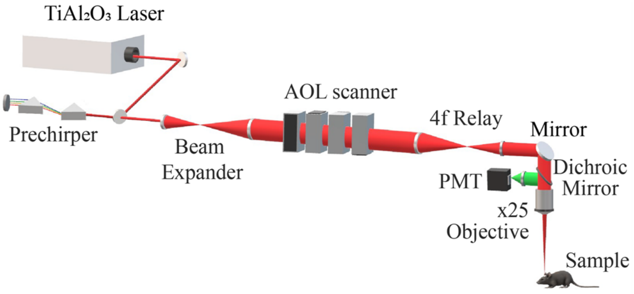

Fig. S2. Optical path of the remote focus acousto-optic lens two-photon microscope. A femtosecond pulsed laser generates a 2 W, 920 nm excitation beam (*red*). A pre-chirper precompensates temporal dispersion introduced by the acousto-optic lens (AOL) scanner. The AOL deflects and adds curvature to the optical beam, enabling remote focusing. A telecentric relay lens system relays the light beam to the back focal plane of the objective lens. Light emitted by two-photon fluorescence excitation (*green*) at the remote focus within the sample is sent back through the objective lens to a photomultiplier tube (PMT) for data acquisition.

immersion objective (Leica HC FLUOTAR L 25X/0.95 W VISIR), giving an excitation NA of approx. 0.57. A two-channel detection system consisting of a dichroic mirror (575dcxr, Chroma Inc.) and emission filters (HQ 525/70m – 2P and HQ 630/100, - 2P), directed the red and green emission of the excited fluorophores onto two PMTs. Green fluorescence was detected with a GaAsP PMT (H7422, Hamamatsu, Japan) and red fluorescence with either a standard PMT (R9880U – 20, Hamamatsu, Japan) or a GaAsP PMT (H7422, Hamamatsu, Japan). The output signals from the PMTs were amplified using 200 MHz preamplifiers (Series DHPVA 100/200 MHz, FEMTO) and processed by a field programmable gate array (FPGA) based acquisition system. The acquisition system consisted of a high-speed ADC (800 MHz, dual channel, NI-5772) and an FPGA (NI FlexRIO, 7966R). A custom-designed FPGA-based control system generated the acoustic frequencies that drive the controlled operation of the AOL scanner. The FPGA AOL control system consisted of a Xilinx VC707 card and a Texas Instruments DAC card (DAC5672EVM). The commands to control the loading and execution of the acoustic drive frequencies were generated by a PC and encoded as RAW Ethernet packets before being transmitted to the AOL controller via a Gigabit Ethernet interface. The AOL control FPGA used an on-chip, direct digital synthesizer to generate the specified acoustic frequency chirps, which were executed upon receiving a start trigger from the data acquisition system. The synthesized digital waveforms were converted into analog signals by the DAC card and amplified by four RF amplifiers before being fed into the AODs. Further details on the design and operating principles of the compact AOL scanning remote focus two-photon microscope can be found in [1,2,5]

S3. Calibration strategies to correct for non-telecentric misalignment

The distortion calibration method we propose in this study used a layer of fluorescent beads to characterize the field distortion for each part of the FOV. During this calibration procedure, the image obtained at the natural focal plane was compared with an image obtained using remote focus. A mismatch between these two images caused by non-telecentricity in the optical path was then precompensated. The values for X_{ERROR}, Y_{ERROR} and Z_{ERROR} were iteratively adjusted until a perfect registration between the predicted focus for a perfectly telecentric system (equivalent to the mechanical focus) and remote focus system was found. This calibration process was fast (a few minutes) and for the case of the AOL microscope, recalibration was only required if optical misalignments develop following movements of components in the optical path.

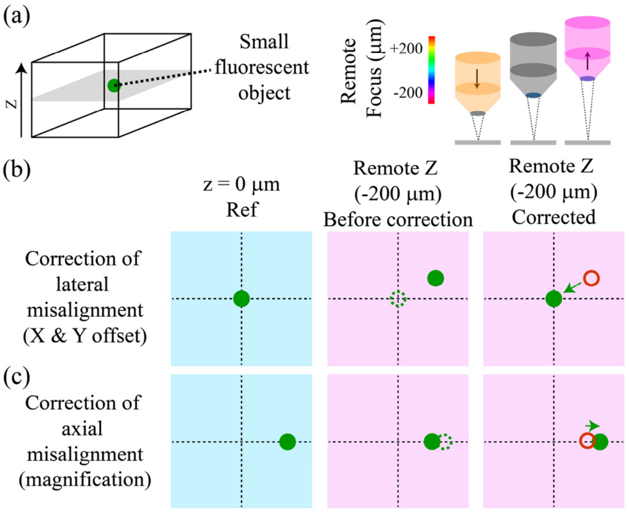

Fig. S3. Schematic of lateral and axial misalignment estimation using only a single bright object. a) *Left*: a small, bright object (*green*) used for the measurement is brought to the natural plane (*gray*). *Right:* the stage is moved mechanically, and the movement is compensated using remote focusing to keep the object in focus. **b)** Lateral misalignment is corrected first. *Left:* the object (*green*) is brought to the center of the FOV at $z = 0$. *Center:* The z-stage is then moved up by 200 µm and compensated by remote focusing -200 µm down (*pink* plane). If the object is shifted laterally compared to its expected position (*green dotted* circle), X_{ERROR} and Y_{ERROR} must be adjusted. *Right:* after successful correction, the object stays at the same xy location when remote focusing. **c)** Correction of axial misalignment. *Left:* the object is brought close to the edge of the FOV at $z = 0$. *Center:* The z-stage is then moved up by 200 µm and compensated by remote focusing -200 µm down (*pink* plane). If the object is shifted laterally compared to its expected position (*green dotted* circle), this indicates a z-dependent magnification that can be corrected by adjusting Z_{ERROR}. *Right:* same as (b).

In practice (i.e. during an experiment), we did not need to re-evaluate the complete field distortion, but only verify that the introduction of additional optical elements (e.g. a coverslip) did not introduce additional distortions. In our experiments, (X_{ERROR}, Y_{ERROR}, Z_{ERROR}) could be easily measured in two steps, using a single fluorescent reference object in the sample (e.g. a small neuron or an injected fluorescent bead in a mouse brain) [Fig. S3]. Compensation of lateral misalignment must be done first. *C-z*-stack measurements of a reference point at the center of the FOV was used to calibrate and compensate lateral misalignment. Once lateral misalignment was corrected, moving the reference to the edge of the FOV could be used to calibrate magnification distortion and therefore compensate axial misalignment. For large axial misalignments, when the object may become out of focus, a manual adjustment can be used. Correcting axial misalignment based on the measured magnification distortion automatically corrects for out-of-focus errors [Fig. 4(g,h)].

S4. Remote focus distortions due to non-telecentricity

The main prediction of the paraxial distortion model is that any deviation of the output of the remote focus unit from its ideal telecentric position, results in a distorted FOV when focusing to any z-plane above or below the objective's natural focus. Fig. S4 and S5 shows the shape of the FOV (pink shaded volume), as predicted by the distortion model (i.e. eqs. S2, S7, S8) in MATLAB. In this example, the system consisted of an 8 mm focal length objective lens, a set of relay lenses of 0.6X magnification and a beam of wavefront curvature κ ranging between ±1 m^{-1} and semi-scan angles (θ_x,θ_y) = (5,5) mrad. In Fig. S4 a lateral misalignment of the wavefront shaping unit of (X_{ERROR}, Y_{ERROR}, Z_{ERROR}) = (2,-2,0) mm, resulted in a skew distortion of the FOV. In Fig. S5, an axial misalignment of (X_{ERROR}, Y_{ERROR}, Z_{ERROR}) = (0,0,100) mm introduces a depth-dependent lateral magnification to the FOV, together with unequally spaced z-focal planes. A

large Z_{ERROR} was chosen for illustrative purposes. To further test the predictions of the remote focus distortion model, a separate paraxial ray model describing the AOL scanner and the subsequent focusing optics of the AOL microscope was developed in MATLAB.

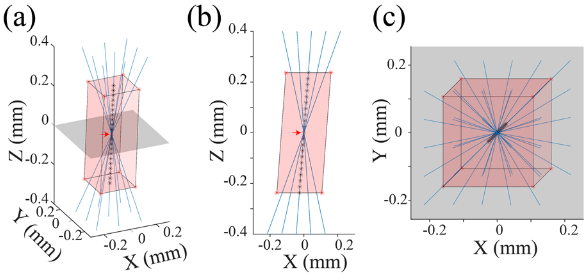

Fig. S4. Predicted effect of lateral misalignment on the 3D FOV. Ray model and distortion model for AOL misalignment (X_{ERROR},Y_{ERROR}, Z_{ERROR}) = (2,-2,0) mm. **a)** 3D view of the FOV predicted by the distortion model (*pink*), overlaid with the rays of the ray model (*blue*) focusing at (*x*,*y*,*z*) = (0,0,0) µm (*red* arrow). Black asterisks indicate the center of the FOV at various remote focus planes. *Gray* plane perpendicular to the FOV indicates the natural focal plane of the objective ($z = 0$). Red asterisks indicate the foci produced by the ray model at the corners of the 3D FOV and match the corners of the pink FOV produced by the distortion model. **b)** same as (a) for the side view. **c)** same as (a) for the top view. Note the skew distortion arising from the lateral misalignment of the AOL.

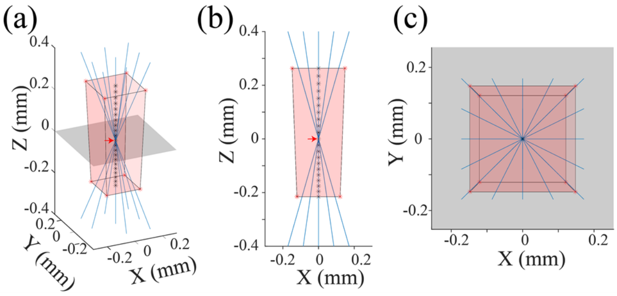

Fig. S5. Predicted effect of axial misalignment on the 3D FOV. Ray model and distortion model for AOL misalignment (X_{ERROR},Y_{ERROR}, Z_{ERROR}) = (0,0,100) mm. **a)** 3D view of the objective FOV predicted by the distortion model (*pink*), overlaid with the rays of the ray model (*blue*) focusing at (*x*,*y*,*z*) = (0,0,0) µm (*red* arrow). *Black* asterisks indicate the center of the FOV at various focus planes. *Gray* plane perpendicular to the FOV indicates the natural focal plane of the objective ($z = 0$). *Red* asterisks indicate the foci produced by the ray model at the corners of the 3D FOV and match the corners of the *pink* FOV produced by the distortion model. **b)** same as (a) for the side view. **c)** same as (a) for the top view. Note varying *z*-plane spacing and varying *xy* plane magnification arising from the axial AOL misalignment.

The acoustic drive equations used in the microscope control software were included in the ray model to simulate the remote focusing operation of the AOL for a 15 mm input beam of 920 nm wavelength. The modelled rays then pass through a relay of 0.6X magnification before being incident on a paraxial model of the Leica objective with a back-aperture diameter of 16 mm and focal length of 8 mm. The focus within the FOV below the objective was found by the least squares intersection point of the rays or by taking the point of maximum intensity of the point spread function given by the Fourier transform of the wavefront error in the iris plane of the objective. The former method was used in all figures showing the distorted bead trajectories predicted by the ray model and is sufficient for calculating the position of the distorted *xy* focus. The Fourier-based method was used in section S5 to predict the out-of-focus error resulting from 152 mm of Z_{ERROR}, when a more accurate *z*-focal position was required, however at the expense of increased computation time. Based on the AOL drive equations, the program could be set to simulate a

volumetric remote focus raster scan and the shape of the FOV could be found by determining the extent of the set of foci produced during the volumetric scan. The position of the AOL unit in the ray model could be misaligned, resulting in a distorted FOV. In Fig. S4 and S5, the blue rays of the ray model are overlaid with the FOV predicted by the distortion model and are shown forming a focus at $(x,y,z) = (0,0,0)$ µm. The black asterisks indicate the foci produced at the center of the FOV for increments of 0.1 m^{-1} in the wavefront curvature κ. The foci of the ray model at the 3D extreme of the simulated raster scan, shown by the red asterisks in Fig. S4 and S5, coincide with the distorted FOV predicted by the distortion model.

The ray model was also used to simulate the operation of the distortion correction scheme by modelling the adjustments to the acoustic drive frequencies required for precompensation. Fig. S6 shows the ray foci produced by the ray model within the objective FOV with distortion precompensation for an AOL misalignment of (X_{ERROR}, Y_{ERROR}, Z_{ERROR}) = (2,-2,100) mm. The pink shaded FOV is the FOV predicted by the distortion model for a telecentric AOL (i.e. (X_{ERROR}, Y_{ERROR}, Z_{ERROR}) = (0,0,0) mm). Fig. S6 demonstrates that the distortion precompensation scheme, which is based on the inverse paraxial distortion model, can be used to correct for remote focus distortions in any optical setup described by a varying wavefront curvature, a paraxial objective of any focal length and, optionally, a set of relay lenses. The ray model can also be used to estimate the real misalignment of the AOL in the microscope by matching the experimentally measured and the theoretically predicted distortion. This feature can be applied to any remote focus microscope provided a *C*-*z*-stack can be obtained. The estimate of the non-telecentric misalignments in the system given by the ray model can be used to physically align the remote focus components to compensate for the observed distortions.

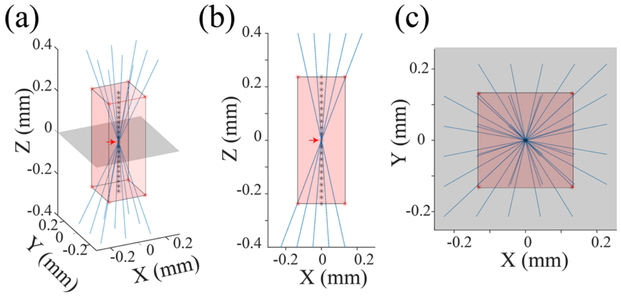

Fig. S6. Correction of predicted axial and lateral misalignment using distortion precompensation. Ray model and distortion model for AOL misalignment (X_{ERROR}, Y_{ERROR}, Z_{ERROR}) = (2,-2,100) mm with distortion precompensation. **a)** 3D view of the FOV predicted by the distortion model (*pink*, using X_{ERROR}, Y_{ERROR}, Z_{ERROR} = (0,0,0) mm), overlaid with the rays of the precompensated ray model (*blue*) focusing at $(x,y,z) = (0,0,0)$ µm (*red* arrow). *Black* asterisks indicate the center of the FOV at various remote foci. Gray plane perpendicular to the FOV indicates the natural focal plane of the objective ($z = 0$). *Red* asterisks indicate the foci produced by the ray model at the corners of the 3D FOV and match the corners of the pink FOV produced by the distortion model. **b)** same as (a) for the side view. **c)** same as (a) for the top view. Note that distortions observed in figures S3 and S4 are corrected but that the central ray remains skewed.

Fig. S7 shows the experimentally measured distortions due to the lateral (S4) and axial (S5) misalignments of the AOL. From this, the AOL misalignment inherent to our remote focus microscope, (X_{ERROR_AOL}, Y_{ERROR_AOL}, Z_{ERROR_AOL}), was estimated to equal (2.3, 1.3, 69) mm.

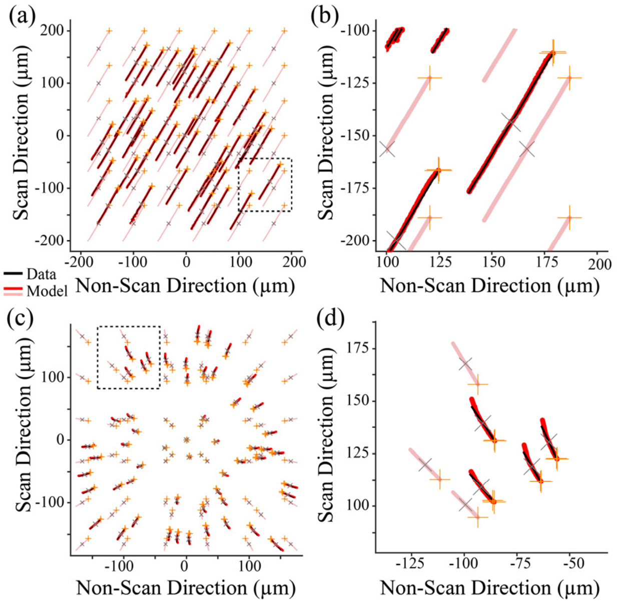

Fig. S7. Experimentally measured and predicted lateral and axial misalignment. a) Measured bead position trajectories (*black*) and bead positions predicted by the ray model at the same location (*red*) and in a grid pattern (*pink*), due to inherent lateral misalignment of our AOL microscope. *Orange* and *gray* asterisks indicate values for +200 µm remote focusing and natural plane imaging, respectively. Axial precompensation was applied to isolate the effect of lateral misalignment. **b)** Zoomed view of the 100 x 100 µm area are indicated in (a) **c)** same as (a) showing inherent axial misalignment of our AOL microscope. Lateral precompensation was applied to isolate the effect of axial misalignment. **d)** Zoomed view of the 100 x 100 µm area are indicated in (c).

S5. Testing the distortion correction scheme

To assess the performance of the distortion correction scheme for axial misalignments of the remote focus unit, the lenses within the relay subsequent to the AOL scanner were modified in order to change the magnification of the optical path and in this way introduced an effective change of the axial misalignment of the AOL unit. By placing a lens of -750 mm and +750 mm focal length directly after the 250 mm focal length lens in the relay, the effective focal length of the first relay lens was changed to 375 mm and 188 mm, respectively. In addition to changing the magnification of the optical system subsequent to the AOL, this introduced an extra divergence and convergence to the input incident on the back aperture of the objective, thus shifting the absolute focal plane. Fig. S8 shows the effect of changing the effective focal length of the first relay lens in the relay. By comparing the experimentally observed distortion to that predicted by the ray model, it is estimated that the addition of the -750 mm *f* and +750 mm *f* lens introduced an effective additional axial misalignment of the AOL equal to +83 mm and -60 mm, respectively.

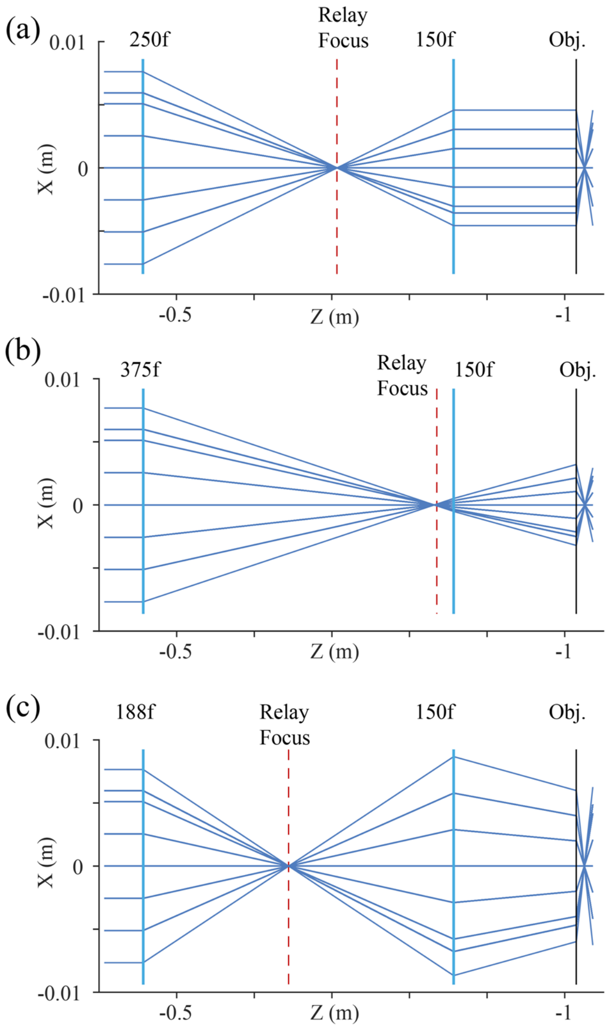

Fig. S8. Ray model diagrams of the modified relay for a collimated AOL output. a) The regular relay (with 2 relay lenses, 250 *f* and 150 *f*, giving 0.6X magnification) present in our AOL microscope. **b)** same as (a), adding a -750 mm focal length lens after the first relay lens, akin to having a single 375 *f* lens. This introduces an additional beam convergence (magnification = 0.4X). **c)** same as (a), adding a +750 mm focal length lens after the first relay lens, akin to having a single 188 *f* lens. This introduces an additional beam divergence (magnification = 0.8X).

To confirm that the distortion pre-compensation scheme, which corrects for the magnification distortion introduced by axial misalignments of the AOL also corrects for the non-constant *z*-spacing, the *z* focal plane position was measured before and after correction by the distortion precompensation scheme [Fig. 4g,h], as described in the main text. The out-of-focus error arising from the pre-existing axial AOL misalignment of 69 mm was measured to be -9.5 µm and 13.5 µm for a remote focus of -200 µm and 200 µm, respectively, which was consistent with the error predicted by the Fourier-based ray model, -10.0 µm and 13.9 µm. With the distortion precompensation scheme enabled, the out-of-focus error was reduced to -1.5 µm and 1.5 µm, respectively. For an axial AOL misalignment of 152 mm (the case shown in Fig. 4 (a,c,e), the out-of-focus error predicted by the Fourier-based ray model for a remote focus of [-200, 200] µm was found to be [-23.5, 32.5] µm.

A schematic of the optical setup used to test the performance of the distortion correction scheme for effective lateral misalignments of the AOL, is given in Fig. S9. A pair of Risley prisms were placed within the relay preceding the objective lens, to mimic lateral displacements of the AOL scanner. The amount of beam displacement introduced by the Risley prisms could be calculated as a function of their rotational position. *C*-*z*-stacks were then collected with and without the correction compensation scheme enabled. The lateral beam displacement by the Risley prisms lead to a skew distortion of the FOV, which could be measured by taking the inverse tangent of the slope of the bead trajectories across a *C*-*z*-stack. The effectiveness of the distortion correction scheme was determined by comparing the experimentally measured skew in the FOV with and without the correction system enabled. As described in section 3 of the main text, the distortion correction scheme is shown to be effective for lateral misalignments of the remote focus unit of up to 6.8 mm.

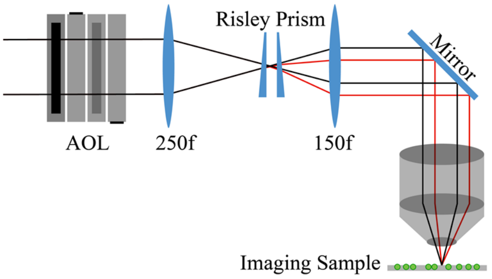

Fig. S9. Schematic of optical setup for introducing different degrees of lateral AOL misalignment. Telecentric condition, where the light beam is centered in the absence of Risley prisms (*black lines*). Light beam when a pair of Risley prisms are placed within the telecentric relay preceding the objective, mimicking a lateral misalignment of the remote focusing device (*red*). This causes a lateral displacement of the beam on the back aperture of the objective and a skew distortion to the imaging FOV.

S6. Applications of the distortion correction scheme

The high precision of the distortion precompensation scheme allowed us to correct for residual second order distortions that could only be observed after distortion precompensation for non-telecentricity in the AOL microscope. This second order distortion was found to vary in a non-uniform manner across the FOV and to increase with longer line scan times. Fig. S10(a) shows the distorted trajectories of 5 µm fluorescent beads across a 270 x 270 x 400 µm FOV, measured with a 200 ns pixel dwell time for a 512x512 pixel scan. The origin of this distortion was investigated using the ray model and was found to arise from a non-parallel input beam to the AOL. Figs. S10(a-c) show the match between the experimental trajectories and those predicted by the ray model for a modelled diverging AOL input of -0.1 m^{-1} curvature. This prediction allowed us to realign the optics preceding the AOL. Figure S10(d) shows the trajectories of the beads in (a), after realignment of the AOL input beam. This resulted in a mean positional error of <0.5 µm over a ± 200 µm remote focus range, independent of pixel dwell time, where the distortion error was calculated as the maximum absolute displacement of the bead trajectory from its position at $z = 0$ in the outer regions of the FOV [S10(e)]. Fig. S10(f) gives the precision achieved over a range of dwell times and remote focus ranges. The slight rise in error at the higher dwell times for the ± 200 µm remote focus range was caused by an AOL aperturing effect that arises when the AODs within the AOL reach their frequency limits for efficient diffraction. This affected the outer regions of the FOV for longer dwell times at increasing *z* focus. For this reason, the mean error against dwell time is also given for a ±100 µm *z* range, showing that realignment of the AOL input beam resolved the dwell time dependence of the residual distortion. This correction highlights that the high precision of the distortion

precompensation scheme allows smaller, higher order distortions to be identified and corrected. The correction for beam divergence into the AOL was only possible after precompensation for the distortion arising from system non-telecentricity.

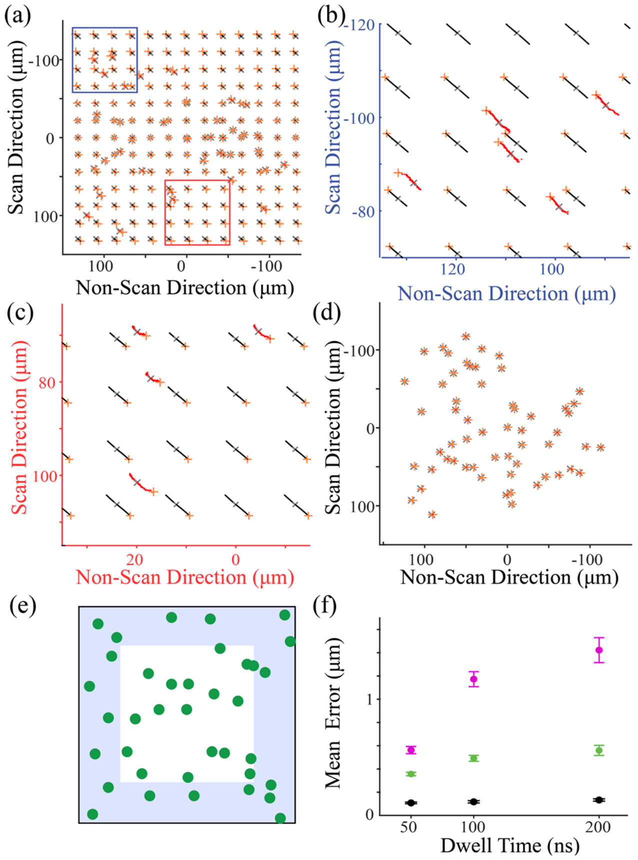

Fig. S10. Properties of residual distortion in AOL FOV and its correction. a) Trajectories of 5 µm fluorescent beads (*red*) over a ±200 µm remote focus range, after distortion precompensation for AOL misalignment. Taken at a 200 ns pixel dwell time for a 512 x 512 pixel FOV. Distortion predicted by the ray model (*black*), for an AOL input beam divergence of -0.1 m^{-1}. *Orange* and *gray* crosses indicate values for 200 µm remote focusing and natural plane imaging, respectively, for both the experimental and theoretical data. **b)** Zoom of the region in (a) marked by the *blue* outline with experimental (*red*) and modelled (*black*) trajectories. **c)** Zoom of the region in (a) marked by the *red* outline. **d)** Trajectories of the beads in (a) after realignment of the AOL input beam with zero divergence. **e)** Illustration of the Region of the FOV where the field distortion is greatest (blue shading, which corresponds to the outer 50%). Beads outside of this region were excluded from the mean distortion error calculation. **f)** Mean positional error of 5 µm bead trajectories in a 270 x 270 µm FOV against dwell time, before (*purple*) and after (*green*) realignment of the AOL input beam for a ±200 µm remote focus range and for a ±100 µm remote focus range (*black*). Error bars show standard error.

References

1. P. A. Kirkby, K. M. N. S. Nadella, and R. A. Silver, "A compact acousto-optic lens for 2D and 3D femtosecond based 2-photon microscopy," Opt Express **18**, 13720 (2010).
2. K. M. N. S. Nadella, H. Roš, C. Baragli, V. A. Griffiths, G. Konstantinou, T. Koimtzis, G. J. Evans, P. A. Kirkby, and R. A. Silver, "Random-access scanning microscopy for 3D imaging in awake behaving animals," Nat Methods **13**, 1001–1004 (2016).

3. G. D. Reddy and P. Saggau, "Fast three-dimensional laser scanning scheme using acousto-optic deflectors," J Biomed Opt **10**, 064038-064038–10 (2005).
4. G. D. Reddy, K. Kelleher, R. Fink, and P. Saggau, "Three-dimensional random access multiphoton microscopy for functional imaging of neuronal activity," Nat Neurosci **11**, 713–720 (2008).
5. G. J. Evans, P. A. Kirkby, K. M. N. S. Nadella, B. Marin, and R. A. Silver, "Development and application of a ray-based model of light propagation through a spherical acousto-optic lens," Opt Express **23**, 23493–23510 (2015).
